# Bimekizumab as treatment for moderate to severe chronic plaque psoriasis: Real world clinical experience from a district general hospital setting

**DOI:** 10.1002/ski2.418

**Published:** 2024-06-27

**Authors:** Omowunmi Ashaolu, Tracy Smith, Amie Askins, Suchitra Rajan, Vincent Li, Sandeep Kamath, Jenny Hughes, Geraldine Haebich

**Affiliations:** ^1^ Department of Dermatology Princess of Wales Hospital Bridgend UK

Dear Editor,

Targeted biologic therapies have transformed the treatment of psoriasis.^1^ The efficacy and safety results with monoclonal antibodies targeting IL‐17RA (brodalumab) and IL‐17A (ixekizumab and secukinumab) validate IL‐17 as an effective therapeutic target for the treatment of plaque psoriasis.^2^ Bimekizumab is a monoclonal IgG1 antibody that selectively inhibits IL‐17A and IL‐17F and was approved by National Institute for Health and Care Excellence in September 2021 for treating adults with moderate to severe plaque psoriasis after standard therapy was unsuccessful or not tolerated.

Bimekizumab has demonstrated efficacy and a good safety profile in phase III clinical trials,^3^ but data from real‐world clinical experience remains limited.

The aim of this study was to evaluate our experience of bimekizumab as a treatment option for patients with moderate to severe chronic plaque psoriasis in a district general hospital setting.

Data collection was carried out retrospectively from January 2022 to October 2022 and included all patients receiving bimekizumab for the treatment of psoriasis. Nine patients were included, 2 males and 7 females with a mean age of 56.7 years (SD 10.4). Scalp psoriasis was present in 5 patients, 1 patient had palmoplantar psoriasis and 6 had psoriatic arthritis. Cardiovascular disease was present in 8 patients. At baseline, 3 patients had a Psoriasis Area Severity Index (PASI) of >10 (median PASI 8.15, range 5.1–17), 6 patients had a Dermatology Life Quality Index (DLQI) >10 (median DLQI 16.5, range 5–28), PASI and DLQI was not recorded in one patient prior to commencing treatment.

All patients had been treated with at least one systemic agent (7 patients with oral methotrexate and 5 with Apremilast) and a biologic prior to commencing bimekizumab (IL 17A inhibitor in 6, IL23 inhibitor in 8 and IL12/23 inhibitor in 2 patients). Bimekizumab was initiated after a mean of 2.8 ± 1.4 and previous biologics lost efficacy over a mean period of 7.2 ± 2.0 years. Treatment of previous biologic agents was stopped due to the loss of efficacy in all the patients.

After 16 weeks of treatment, all patients achieved PASI 75 (median PASI 0, range 0–6.4) and a 5‐point reduction in DLQI <10 (median DLQI 0, range 0–4). There was also improvement with scalp psoriasis, palmoplantar psoriasis and joint symptoms. None of the patients experienced any adverse effects with Bimekizumab.

Bimekizumab appears to be effective in clearing psoriasis including hard‐to‐treat sites with improvement in the quality of life. This case series was limited by small patient numbers, and more real‐world experience using bimekizumab is needed.

## CONFLICT OF INTEREST STATEMENT

None to declare.

## AUTHOR CONTRIBUTIONS

**Omowunmi Ashaolu**: Formal analysis (equal); writing – original draft (equal); writing – review & editing (equal). **Tracy Smith**: Data curation (equal). **Amie Askins**: Data curation (equal). **Suchitra Rajan**: Writing – review & editing (equal). **Vincent Li**: Writing – review & editing (equal). **Sandeep Kamath**: Writing – review & editing (equal). **Jenny Hughes**: Writing – review & editing (equal). **Geraldine Haebich**: Conceptualization (equal); writing – review & editing (equal).

## ETHICS STATEMENT

Not applicable.

## PATIENT CONSENT

Not applicable.

## Data Availability

The data underlying this article will be shared on reasonable request to the corresponding author.
